# Assessing the Pathogenicity, Penetrance, and Expressivity of Putative Disease-Causing Variants in a Population Setting

**DOI:** 10.1016/j.ajhg.2018.12.015

**Published:** 2019-01-18

**Authors:** Caroline F. Wright, Ben West, Marcus Tuke, Samuel E. Jones, Kashyap Patel, Thomas W. Laver, Robin N. Beaumont, Jessica Tyrrell, Andrew R. Wood, Timothy M. Frayling, Andrew T. Hattersley, Michael N. Weedon

**Affiliations:** 1 Institute of Biomedical and Clinical Science, University of Exeter Medical School, Research, Innovation, Learning and Development building, Royal Devon & Exeter Hospital, Barrack Road, Exeter EX2 5DW, UK

**Keywords:** genotyping, rare variant, SNP-chip, pathogenicity, penetrance, biobank, genetic, variant

## Abstract

More than 100,000 genetic variants are classified as disease causing in public databases. However, the true penetrance of many of these rare alleles is uncertain and might be over-estimated by clinical ascertainment. Here, we use data from 379,768 UK Biobank (UKB) participants of European ancestry to assess the pathogenicity and penetrance of putatively clinically important rare variants. Although rare variants are harder to genotype accurately than common variants, we were able to classify as high quality 1,244 of 4,585 (27%) putatively clinically relevant rare (MAF < 1%) variants genotyped on the UKB microarray. We defined as “clinically relevant” variants that were classified as either pathogenic or likely pathogenic in ClinVar or are in genes known to cause two specific monogenic diseases: maturity-onset diabetes of the young (MODY) and severe developmental disorders (DDs). We assessed the penetrance and pathogenicity of these high-quality variants by testing their association with 401 clinically relevant traits. 27 of the variants were associated with a UKB trait, and we were able to refine the penetrance estimate for some of the variants. For example, the HNF4A c.340C>T (p.Arg114Trp) (GenBank: NM_175914.4) variant associated with diabetes is <10% penetrant by the time an individual is 40 years old. We also observed associations with relevant traits for heterozygous carriers of some rare recessive conditions, e.g., heterozygous carriers of the ERCC4 c.2395C>T (p.Arg799Trp) variant that causes Xeroderma pigmentosum were more susceptible to sunburn. Finally, we refute the previous disease association of *RNF135* in developmental disorders. In conclusion, this study shows that very large population-based studies will help refine our understanding of the pathogenicity of rare genetic variants.

## Introduction

One of the ongoing challenges in genetic medicine is that of variant interpretation. Many variants and genes have been erroneously associated with disease as a result of study design problems (including ascertainment bias and inadequate cohort size),^1^, ^2^, ^3^ as well as biological phenomena such as genetic heterogeneity, reduced penetrance, variable expressivity, composite phenotypes, pleiotropy, and epistasis.^4^, ^5^, ^6^, ^7^, ^8^, ^9^, ^10^, ^11^, ^12^, ^13^ These issues have resulted in ambiguity over how to interpret clinically ascertained variants found in individuals with no known family history or symptoms of the disease.^14^ Although there has traditionally been a division between rare disease genetics (studied in small disease cohorts and individual high-risk families) and common disease genetics (studied in large disease cohorts and population biobanks), in reality a continuum of causality is likely for many human disorders.^15^ Fortunately, rare and common disease studies suffer from opposing ascertainment biases. Clinical and family-based cohorts ascertained as a result of a specific clinical presentation will tend to overestimate the penetrance of any identified disease-causing variants.^16^ In contrast, population cohorts tend to be enriched for healthy individuals (the so-called “healthy volunteer” selection bias) who have both the time and ability to volunteer for a study,^17^, ^18^ and they will therefore tend to underestimate penetrance. Population cohorts that have high-resolution genetic and clinical data are therefore invaluable for establishing minimum penetrance estimates, exploring variable expressivity, and challenging pathogenicity assertions made in the clinical arena.

Several studies have already started to bridge this gap by using population data to evaluate rare disease-causing variants,^19^, ^20^ refine penetrance estimates,^21^ and refute reportedly pathogenic variants.^22^, ^23^ These previous studies were mostly limited to a very specific set of variants (e.g. protein-truncating variants) or one particular disease, or they were too small to statistically test phenotypic penetrance. With its wealth of linked phenotypic and clinical information on ∼500,000 genotyped individuals, UK Biobank (UKB)^24^ offers a powerful dataset in which to systematically evaluate the pathogenicity, penetrance, and expressivity of clinically important variants in the population. However, differences in the technologies used for assaying genetic variation can hinder these analyses. A particular concern is the use of genotyping arrays (such as those currently used by UKB),^25^ which have been designed primarily to assay common variation. In contrast, rare single-nucleotide variants (SNVs) and small insertions/deletions (indels) have typically been detected through sequencing assays.^26^ A method is therefore needed to select well-genotyped, rare variants in UKB; this method can then be used in addressing biological and clinical questions.

Here we describe a systematic method for evaluating the analytical validity of rare-variant genotyping data from the UKB arrays, investigate the relationship between data quality and minor-allele frequency (MAF), and evaluate the association of a subset of clinically interesting, well-genotyped coding variants with relevant phenotypes in UKB. We focus on ClinVar variants that have been classified as “pathogenic” or “likely pathogenic” by at least one submitter,^27^ as well as variants in genes known to cause two specific monogenic diseases, maturity-onset diabetes of the young (MODY [MIM: 606391]) and developmental disorders (DDs), in which we have some expertise.

## Subjects and Methods

### UKB Cohort

Between 2006 and 2010, UKB recruited more than 500,000 individuals aged 37–73 years from across the UK. Participants provided a range of information (e.g., demographics, health status, lifestyle) via questionnaires and interviews. Additionally, anthropometric measurements; blood-pressure readings; and blood, urine, and saliva samples were taken for future analysis. Genotypes for SNVs and indels were generated from the Affymetrix Axiom UKB array (∼450,000 individuals) and the UKBiLEVE array (∼50,000 individuals) in 106 batches of ∼4,700 samples. This dataset underwent extensive central quality control (see Web Resources).^25^ We limited our analysis to 379,768 QC-passed white Europeans.

### Variant Prioritization

We annotated variants by using Annovar^28^ and calculated MAFs by using PLINK.^29^ To prioritize variants of potential clinical importance, we selected those with at least one classification of pathogenicity (pathogenic or likely pathogenic) in the ClinVar database;^27^ variants with conflicting classifications were not excluded. In addition, irrespective of their presence in ClinVar, we selected predicted protein-truncating variants (PTVs; stopgain SNVs and frameshift indels) and known pathogenic functional variants (nonsynonymous SNVs and inframe indels) in genes known to be associated with MODY^30^, ^31^ and dominant DD^32^^,^^33^ for detailed evaluation. These diseases and genes were selected on the basis of our own prior experience, the availability of well-curated gene lists that include the mode of inheritance and mechanism of action, and the different prior probabilities associated with finding diabetes (a common disease) and severe DD (a rare disease) in UKB. We excluded common variants (MAF > 1%) because these have already been thoroughly investigated through genome-wide association studies,^34^, ^35^ and we further refined the list of variants to include only those where the Hardy-Weinberg equilibrium (HWE) had a p > 0.05 and the proportion of missing genotypes across all samples was < 0.01 (n = 4,585).

### Assessing Analytical Validity

To assess the analytical validity of these variants, we used Evoker Lite (see Web Resources) to generate cluster plots of intensities, and we combined data from all the batches into one plot for each variant. Cluster plots were manually assessed and ranked in quality from 1–5, where 1 = poor quality, no discernible separate clusters; 2 = poor quality, no discernible separate clusters but noisy data; 3 = unclear or uncertain; 4 = good quality, clearly separable clusters but noisy data; and 5 = good quality, clear separation between clusters (Figure S1). In an initial 750-variant subset that was independently evaluated by two scientists (Figure S2), correlation between the two independent scorers was high (R^2^ = 0.8), and there was a 95% agreement in low quality (score = 1 or 2) versus high quality (score = 4 or 5) variants. All remaining variants of interest were evaluated by one scientist, and those with high quality scores were checked by the second scientist. Only variants with an average score of >4 were retained for further analysis. For all 1,244 high-quality variants, we assessed whether the rare genotype calls were unusually distributed across the 107 genotype batches. None of the rare genotype calls at these variants were entirely due to calls from a single batch. Across the 1,244 variants, the highest proportion of rare genotype calls in a single batch was four from a total of 13 for Affx-89007317. A plot of total allele count for each variant versus maximum allele count across each individual batch demonstrated a linear association with no clear outlying variants.

### Assessing Clinical Relevance

Using PLINK, we ran a phenome-wide association in 379,768 QC-passed white Europeans for all of our 1,244 high-quality rare variants against a curated list of 401 clinically relevant traits in UKB (Table S1)^29^ and those variants with a Bonferroni-corrected p < 1×10^-7^ (0.05/[401^∗^1244]) were prioritized for detailed evaluation. For continuous traits, we used linear regression wherein we adjusted for age, sex (unless the trait was sex-specific), center, genotyping chip, and ten ancestry principal components. For binary traits, we used Fisher’s exact test as the primary association method. We excluded three variants that had been reclassified in ClinVar as benign since our variant annotation (November 2017). To assess the potential clinical implications of high-quality rare variants, we compared the UKB traits with the clinical presentation of the disease for each gene and the evidence supporting the assertion of pathogenicity of the variant by using ClinVar,^27^ DECIPHER,^36^ and OMIM.^37^ For high-quality rare variants in MODY genes and PTVs in DD genes, we had no p-value cut-off for investigating diabetes and developmental traits (cognitive function, educational attainment, body mass index, height, hearing, and albumin creatinine ratios). Conditional analysis of the most-associated regional variant (within a 1 Mb window) from each trait led us to remove one trait-variant association that was explained by linkage disequilibrium with a common causal variant.

## Results

### Variants below 0.001% Frequency Are Not Reliably Genotyped

**Figure 1:**
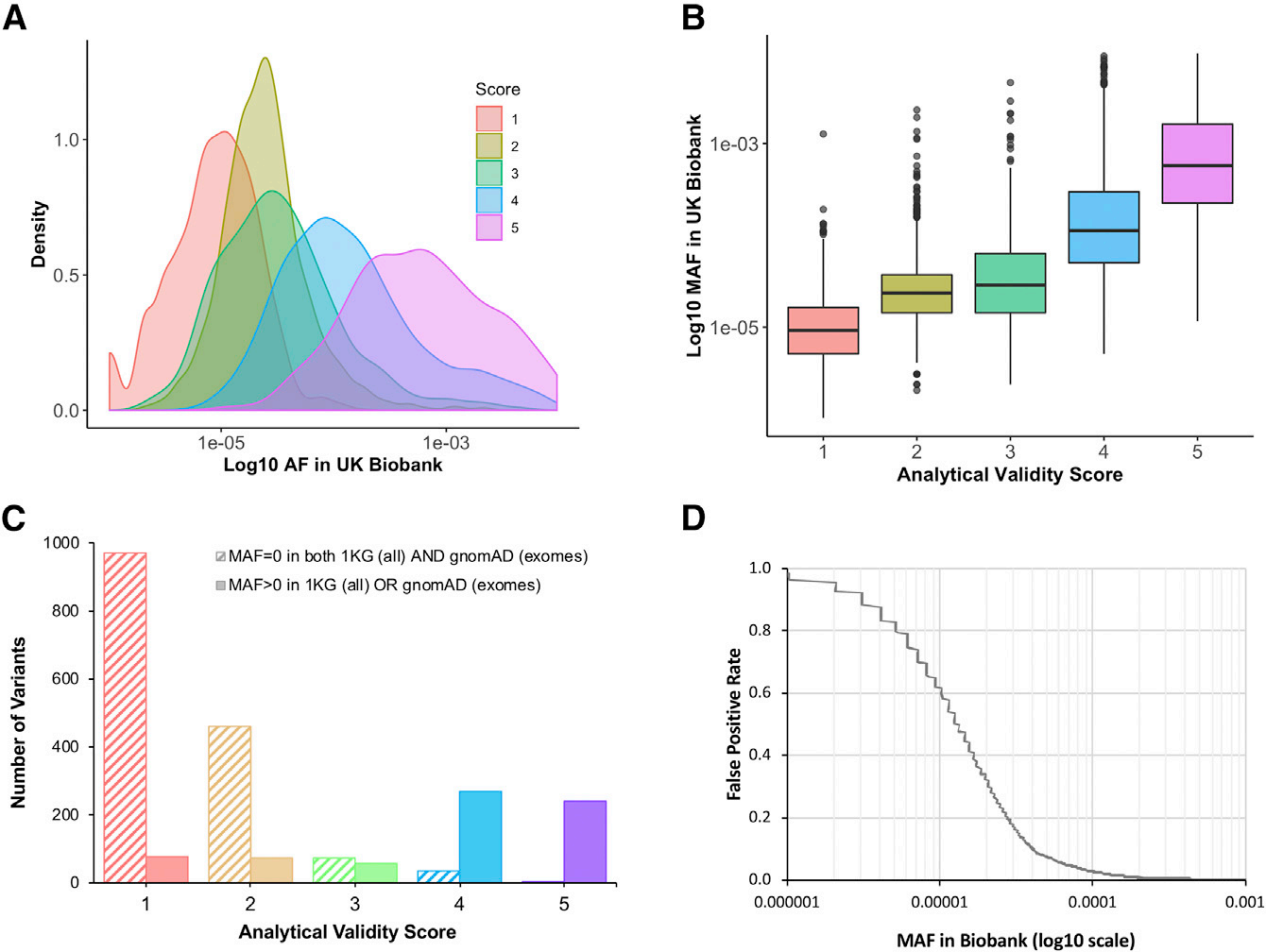
Correlation between Minor Allele Frequency and Analytical Validity Quality Score (A and B) Density plot (A) and boxplot (B) of manual quality scores (from 1–5, see Figure S1) of genotype data in UKB versus minor allele frequency (MAF) for 4,585 putatively clinically important variants, where MAF < 1%, Hardy–Weinberg equilibrium (HWE) > 0.05, and missingness < 0.01. (C) Histogram of the number of variants at each quality score versus presence or absence of the variant in gnomAD (exome data) or the 1000 Genomes Project. Red = score 1; gold = score 2; green = score 3; blue = score 4; purple = score 5. (D) Estimation of the false-positive rate (FPR) versus MAF for variants assayed with the UKB genotyping arrays, calculated by the grouping of quality scores into low (score = 1 or 2) and high (score = 4 or 5) and use of the rocreg command in Stata for fitting a ROC curve.

Across all the variants evaluated for analytical validity via combined cluster plots (n unique = 4,585; see Subjects and Methods), we categorized 27% as high quality (average score >4), 64% as low quality, most likely false positives (average score <2.5), and 9% as unclear (Table 1). There was a strong correlation between the analytical-validity quality score and both the MAF (Table 1 and Figure 1) and the presence of the variant in either gnomAD^38^ or the 1000 genomes project.^39^ For low- versus high-quality variants, a nonparametric regression analysis estimated the area under the ROC curve to be 0.95 (95% confidence interval (CI) = 0.943–0.956); the false-positive rate (FPR) at MAF > 0.005% was ∼20%, but the FPR was ∼60% at MAF > 0.001%.

**Table 1:** Evaluated Variants

**MAF Bin (%)**	**FP**	**TP**	**Unclear**	**Total**
0–0.0005	511	0	11	522
0.0005–0.001	607	8	59	674
0.001–0.005	1,598	218	210	2,026
0.005–0.01	138	204	73	415
0.01–0.05	66	456	48	570
0.05–0.1	2	129	5	136
0.1–0.5	6	189	7	202
0.5–1	0	40	0	40
Total	2,928	1,244	413	4,585

Number of variants manually evaluated for analytical validity in different MAF bins; quality scores are grouped into false positives (FP, score = 1 or 2), unclear scores (score = 3), and true positives (TP, score = 4 or 5).

### Estimates of Minimum Effect Size for Known Pathogenic Variants

The 1,244 high-quality, putatively pathogenic rare variants, along with their ClinVar-associated disease and the allele frequencies in UKB and gnomAD, are shown in Table S2. Of these variants, only 27 were associated (p < 1 × 10^-7^) with one of the 401 traits we tested against in UKB (Table 2 and Table S3). Of these, 13 have previously been linked with a dominant disease, although most are considered to be only risk factors or low-penetrance variants rather than true highly pathogenic monogenic variants. Specifically, we observed well-established associations between variants in *HOXB13*^40^ (MIM: 604607) and *PALB2*^41^ (MIM: 610355) and prostate cancer (MIM: 176807) and breast cancer (MIM: 114480), respectively. The effect sizes are broadly in line with estimates from population-based studies and consistently lower than those of family-based studies (e.g.^40^, ^41^). The HOXB13 c.251G>A (p.Gly84Glu) variant was originally suggested to increase risk 20-fold,^40^ but a population-based study^42^ of this variant reported an odds ratio of 3.5, 95% CI ([2.4, 5.2]), similar to the odds ratio observed in the UKB of 4.09, 95% CI ([3.24, 5.17]). For *PALB2*, a similar loss-of-function variant found at relatively high frequency in Finland had an odds ratio of 11.3 (95% CI ([1.8, 57.81])) when family-based cases were used, but it had an odds ratio of only 3.94, 95% CI ([1.5, 12.1]) when unselected cases were used;^43^ this ratio is comparable to the estimate of 4.55, 95% CI ([3.05, 6.79]) in the UKB. The other 11 variants were causally linked to disease, and we observed that these variants were associated with a related trait in our population-based cohort (Table 2). In *FLG* (MIM: 135940), two PTVs that cause ichthyosis vulgaris^44^ (MIM: 146700) were associated with increased odds of eczema (MIM: 603165) [odds ratios were 1.66 (95% CI [1.40, 1.98] and 1.96 (95% CI [1.69, 2.27])], consistent with effect sizes for loss-of-function mutations from previous studies.^45^ A *TSHR* (MIM: 603372) PTV that causes nonautoimmune hyperthyroidism^46^ (MIM: 609152) was associated with an increased odds of hypothyroidism (odds ratio 3.34, 95% CI [2.47, 4.51]). A nonsynonymous *LRRK2* (MIM: 609007) variant that causes Parkinson disease (MIM: 607060) was associated with an odds ratio of 4.76 (95% CI [3.25, 6.96]) that a person would have a parent with Parkinson disease; this estimate is consistent with studies of affected family members.^47^, ^48^, ^49^ A nonsynonymous *PER3* (MIM: 603427) variant previously classified as pathogenic for advanced sleep phase syndrome (MIM: 616882) had an odds ratio of only 1.38 for being a morning person and advanced sleep timing by only 8 minutes, 95% CI ([4, 13])^50^, ^51^ compared to a reported 4.2 hour shift in midpoint sleep. Height, skeletal weight, and male pattern baldness were negatively associated with two nonsynonymous variants in *AR* (MIM: 313700) that cause partial androgen insensitivity syndrome.^52^ Finally, a nonsynonymous *MYH7* variant (MIM: 160760), which has been classified by a ClinGen expert panel as pathogenic for hypertrophic cardiomyopathy^53^ (MIM: 192600), was associated with a reduced pulse rate of 5 (95% CI ([4, 6])) beats per minute.

**Table 2:** Pathogenic Variants

**Gene**	**UKB ID**	**Position (GRCh37)**	**HGVS**	**MAF White British (%)**	**Significantly Associated Trait(s) in UKB (Units)**	**Odds Ratio or Beta [95% CI]**	**p value**	**Linked Disease (Mode of Inheritance)**
*ACSF3*	dbSNP: rs141090143	chr16: 89220556 C>T	GenBank: NM_174917:c.C1672T:p.R558W	0.632	ease of sunburn (number of episodes)	0.31 [0.20, 0.42]	4 × 10^−10^	combined malonic and methylmalonic aciduria (AR)
*AR*	dbSNP: rs137852591	chrX: 66941751 C>G	GenBank: NM_000044:c.C2395G:p.Q799E	0.129	skeletal mass (SD)	−0.16 [−0.21, −0.11]	1 × 10^−10^	partial androgen insensitivity syndrome (XLR)
					height (cm)	−0.85 [−1.27, −0.43]	1 × 10^−8^	
	dbSNP: rs1800053	chrX: 66931295 C>A	GenBank: NM_000044:c.C1937A:p.A646D	0.269	balding pattern (males only)	−0.13 [−0.17, −0.08]	1 × 10^−8^	partial androgen insensitivity syndrome (XLR)
*ERCC4*	dbSNP: rs121913049	chr16: 14041848 C>T	GenBank: NM_005236:c.C2395T:p.R799W	0.060	ease of sunburn (number of episodes)	0.98 [0.64, 1.33]	2 × 10^−8^	xeroderma pigmentosum (AR)
*FLG*	dbSNP: rs150597413	chr1: 152277622 G>T	GenBank: NM_002016:c.C9740A:p.S3247X	0.369	eczema	1.66 [1.40, 1.98]	9 × 10^−8^	ichthyosis vulgaris (AD)
	dbSNP: rs138726443	chr1: 152280023 G>A	GenBank: NM_002016:c.C7339T:p.R2447X	0.446	eczema	1.96 [1.69, 2.27]	5 × 10^−16^	ichthyosis vulgaris (AD)
*GCK*	dbSNP: rs104894006	chr7: 44189591 G>A	GenBank: NM_000162:c.C556T:p.R186X	0.001	maturity-onset diabetes of the young	68 [14, 325]	2 × 10^−8^	diabetes mellitus (AD)
*HBB*	Affx-52141620	chr11: 5248004 G>A	GenBank: NM_000518:c.C118T:p.Q40X	0.005	mean corpuscular volume (SD)	−2.92 [−3.26, −2.57]	6 × 10^−63^	beta-thalassemia (AR)^∗^
					red blood cell distribution width (SD)	1.87 [1.53, 2.21]	5 x 10^−27^	
*HOXB13*	dbSNP: rs138213197	chr17: 46805705 C>T	GenBank: NM_006361:c.G251A:p.G84E	0.160	prostate cancer	4.09 [3.24, 5.17]	1 × 10^−23^	prostate cancer susceptibility (AD)
					father with prostate cancer	1.75 [1.47, 2.09]	4 × 10^−9^	
*HNF4A*	dbSNP: rs137853336	chr20: 43042354 C>T	GenBank: NM_175914:c.340C>T:R114W	0.015	diabetes	2.9 [1.7, 5]	3 × 10^−4^	maturity-onset diabetes of the young (AD)
*HSPA9*	Affx-80274027	chr5: 137902404 CT>-	GenBank: NM_004134:c.882_883del:p.T294fs	0.017	mean corpuscular volume (SD)	−0.49 [−0.67, −0.32]	2 × 10^−8^	even-plus syndrome (AR)
					red blood cell distribution width (SD)	1.17 [0.99, 1.34]	9 × 10^−40^	
*KLF1*	Affx-80299186	chr19: 12995833 ->C	GenBank: NM_006563:c.954dupG:p.R319fs	0.017	mean corpuscular volume (SD)	−1.27 [−1.45, −1.1]	9 × 10^−48^	blood group Lutheran inhibitor (AD)
					red blood cell distribution width (SD)	1.48 [1.3, 1.65]	2 × 10^−63^	
*LRRK2*	dbSNP: rs34637584	chr12: 40734202 G>A	GenBank: NM_198578:c.G6055A:p.G2019S	0.032	parent with Parkinson disease	4.76 [3.25, 6.96]	1 × 10^−11^	Parkinson disease (AD)
*MYH7*	Affx-86888962	chr14: 23887458 C>T	GenBank: NM_000257:c.C4130T:p.T1377M	0.117	pulse rate (beats per minute)	−4.75 [−5.47, −4.01]	4 × 10^−41^	primary familial hypertrophic cardiomyopathy (AD)
*NPC1*	dbSNP: rs80358259	chr18: 21116700 A>G	GenBank: NM_000271:c.T3182C:p.I1061T	0.075	mean corpuscular volume (SD)	−0.24 [−0.32, −0.15]	2 × 10^−8^	Niemann-Pick disease (AR)
*OCA2*	dbSNP: rs28934272	chr15: 28230247 C>T	GenBank: NM_000275:c.G1327A:p.V443I	0.834	ease of sunburn (number of episodes)	0.49 [0.40, 0.58]	1 × 10^−47^	oculocutaneous albinism (AR)
	dbSNP: rs121918170	chr15: 28228529 T>C	GenBank: NM_000275:c.A1465G:p.N489D	0.094	ease of sunburn (number of episodes)	0.91 [0.64, 1.18]	1 × 10^−14^	oculocutaneous albinism (AR)
*PALB2*	dbSNP: rs180177132	chr16: 23632683 C>T	GenBank: NM_024675:c.G3113A:p.W1038X	0.033	breast cancer	4.55 [3.05, 6.79]	2 × 10^−10^	familial breast cancer (AD)
					mother with breast cancer	2.62 [1.92, 3.59]	5 × 10^−8^	
*PER3*	dbSNP: rs139315125	chr1: 7869960 A>G	GenBank: NM_001289862:c.A1250G:p.H417R	0.438	morning person	1.37 [1.27, 1.47]	2 × 10^−16^	advanced sleep phase syndrome (AD)
	dbSNP: rs150812083	chr1: 7869953 C>G	GenBank: NM_001289862:c.C1243G:p.P415A	0.458	morning person	1.35 [1.25, 1.46]	7 × 10^−15^	
*SEC23B*	dbSNP: rs121918221	chr20: 18496339 G>A	GenBank: NM_006363:c.G325A:p.E109K	0.027	red blood cell distribution width (SD)	0.39 [0.25, 0.52]	3 × 10^−8^	congenital dyserythropoietic anemia (AR)
*SLC6A19*	dbSNP: rs121434346	chr5: 1212453 G>A	GenBank: NM_001003841:c.G517A:p.D173N	0.442	red blood cell distribution width (SD)	−0.15 [−0.18, −0.11]	2 × 10^−16^	neutral 1 amino acid transport defect (AR)
*TACR3*	dbSNP: rs144292455	chr4: 104577415 C>T	GenBank: NM_001059:c.G824A:p.W275X	0.054	reproductive age at menarche (yr)	0.66 [0.45, 0.87]	2 × 10^−10^	hypogonadotropic hypogonadism (AR)
*TMPRSS6*	dbSNP: rs137853120	chr22: 37469593 C>T	GenBank: NM_153609:c.G1561A:p.D521N	0.019	mean corpuscular volume (SD)	−0.67 [−0.83, −0.51]	3 × 10^−16^	microcytic anemia (AR)
					red blood cell distribution width (SD)	0.69 [0.53, 0.85]	5 × 10^−17^	
*TSHR*	dbSNP: rs121908866	chr14: 81610039 G>A	GenBank: NM_000369:c.G1637A:p.W546X	0.041	hypothyroid	3.34 [2.47, 4.51]	7 × 10^−12^	hypothyroidism (AD, AR)
					autoimmune disease	2.31 [1.76, 3.04]	4 × 10^−8^	
*TNFRSF13B*	dbSNP: rs34557412	chr17: 16852187 A>G	GenBank: NM_012452:c.T310C:p.C104R	0.703	mean corpuscular volume (SD)	−0.09 [−0.12, −0.07]	4 × 10^−11^	common variable immunodeficiency (AD, AR)

Reduced penetrance, variable expressivity, and carrier phenotypes for rare (MAF < 1%) ClinVar pathogenic variants with genome-wide significant associations in UKB. Abbreviations are as follows: UKB = UK Biobank, HGVS = Human Genome Variation Society, MAF = minor allele frequency, SD = standard deviations, cm = centimeters, yr = years, CI = confidence interval, AD = autosomal dominant, AR = autosomal recessive, XLR = X-liniked recessive.

### Reduced Penetrance for the Common HNF4A p.Arg114Trp MODY Mutation

**Figure 2:**
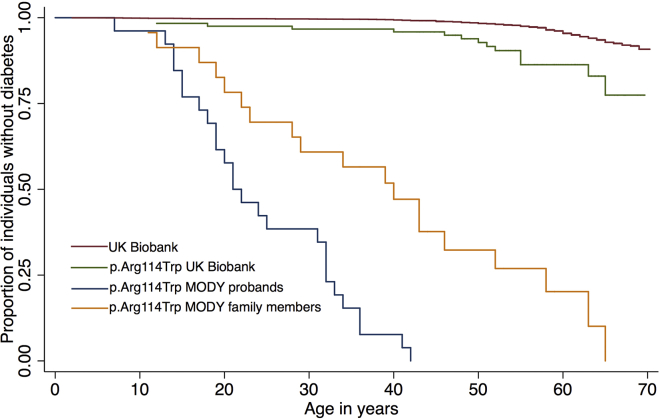
Comparison of Penetrance Estimate for *HNF4A* p.Arg114Trp in UK Biobank versus Previously Published Estimates from MODY Cohort Studies A Kaplan-Meier plot of the proportion of individuals who are diabetes free at various ages for 379,768 individuals from UK Biobank (red line), 122 UK Biobank individuals who are heterozygous for HNF4A p.Arg114Trp (green line), 26 MODY referral probands (blue line), and 24 family members of the probands (yellow line) from Laver *et al.*^54^

We specifically investigated known pathogenic variants and PTVs in MODY genes, where we found two rare variants that were high quality, definitely pathogenic, and strongly associated with diabetes (Table 2): a very rare stop-gain variant in *GCK* (MIM: 138079) (OR = 68, 95% CI [14, 328], p = 2 × 10^-8^), and a nonsynonymous variant (p.Arg114Trp) in *HNF4A* (MIM: 600281) (OR = 2.9, 95% CI [1.7, 5.0], p = 3 × 10^-4^). Both were associated with diabetes in UKB, in line with previous findings.^54^, ^55^, ^56^ However, the penetrance of the *HNF4A* variant was previously estimated on the basis of a large MODY diabetes cohort to be up to 75% at age 40 years,^54^ although we estimate the minimum penetrance to be <10% from UKB (Figure 2). This has important implications for the attributable risk associated with the variant in different cohorts and for the interpretation of genetic test results: if the p.Arg114Trp variant was found in an affected individual after clinical testing, it might still be the primary cause of that person’s diabetes, although incidental discovery of the variant in an unaffected individual would not be predictive.

### Related Mild Heterozygous Phenotypes in Autosomal-Recessive Disorders

Of our 27 high-quality, rare putatively pathogenic variants associated with a trait in UKB, 16 have previously been linked with a recessive disease (Table 2). We observed associations with milder or related traits in the heterozygous carriers of these monogenic recessive diseases in our population cohort. A nonsynonymous *ERCC4* (MIM: 133520) variant that causes recessive xeroderma pigmentosum^57^ and two nonsynonymous *OCA2* (MIM: 611409) variants that cause oculocutaneous albinism^58^, ^59^ (MIM: 203200) were associated with ease of sunburn. A stopgain *TACR3* (MIM: 162332) variant, which causes recessive hypogonadotropic hypogonadism^60^, ^61^ (MIM: 614840), was associated with an 8 month increase in age at which a girl experiences menarche. In addition, variants in six genes known to be associated with different recessive blood-related disorders were also associated with decreased mean corpuscular volume and/or increased red blood cell distribution width (such genes included *HBB* [MIM: 141900], variants in which cause β-thalassemia (MIM: 612985), but the carrier state is already known to cause the much milder β-thalassemia minor^62^).

### Benign Protein-Truncating Variants in Monogenic Genes

We focused our clinical analysis of variants in DD genes on just PTVs, of which six (including two variants in one gene) were of high quality and were in genes that are reported to cause disease via a haploinsufficiency mechanism (Table 3). None of these variants were associated with developmentally relevant traits in UKB (p > 0.1), suggesting they are all benign. For three variants, the location of the variant in the gene is notably different from that of known pathogenic variants. *GNAS* (MIM: 139320) is the only one of the five genes with a high probability of being loss-of-function intolerant (pLI)^38^ on the basis of the frequency of loss-of-function variants in the Exome Aggregation Consortium (ExAC) browser.^38^ The stop-gain variant in *GNAS* is present in the highly variable first exon of the gene and is likely to result in nonsense-mediated RNA decay; in contrast, pathogenic *GNAS* variants that cause Albright hereditary osteodystrophy (MIM: 103580) are located in later, highly constrained exons.^63^ Similarly, the stop-gain variant in *TGIF1* (MIM: 602630) is located in the first exon, where multiple PTVs in gnomAD^38^ are also located, but *TGIF1* pathogenic variants causing holoprosencephaly are located in the final exons, where they affect DNA binding affinity.^64^ Finally, a frameshift deletion in *HIST1H1E* (MIM: 142220) is located near the start of the single exon of this gene; however, pathogenic *HIST1H1E* frameshift deletions that cause child overgrowth and intellectual disability are located near the end of the exon, where they result in a truncated histone protein with lower net charge that is less effective at binding DNA.^65^ Hence, we believe that these three rare PTVs are benign because of their locations, despite the fact that they occur in genes that cause dominant DD via haploinsufficiency.

**Table 3:** Benign Variants

**Gene**	**UKB ID**	**Position (GRCh37)**	**HGVS**	**MAF (%)**	**Biobank Trait**	**Beta [95% CI]**	**p Value**	**Linked Monogenic Disease**
*COL4A3*	Affx-80270894	chr2: 228148945 G>GAGTAAAGGGCC	GenBank: NM_000091:c.2766_2776del:p.G922fs	0.01813	education years	−0.007 [−0.17, 0.156]	0.93	DD (Alport syndrome,
					fluid intelligence	−0.085 [−0.3, 0.133]	0.45	autosomal dominant)
					BMI	0.096 [−0.07, 0.259]	0.25	
					height	−0.097 [−0.26, 0.066]	0.24	
					albumin creatine ratio	0.941 [0.44, 2.015]	0.88	
					hearing left	−0.13 [−0.41, 0.144]	0.35	
					hearing right	0.076 [−0.2, 0.35]	0.59	
*GNAS*	dbSNP: rs200910410	chr20: 57428858 T>C	GenBank: NM_080425:c.C538T:p.Q180X	0.03063	education years	−0.053 [−0.18, 0.072]	0.41	DD (Albright hereditary
					fluid intelligence	0.062 [−0.12, 0.239]	0.49	osteodystrophy)
					BMI	0.048 [−0.08, 0.174]	0.46	
					height	−0.105 [−0.23, 0.021]	0.10	
*HIST1H1E*	Affx-89024826	chr6: 26156672 T>TC	GenBank: NM_005321:c.55delC:p.P19fs	0.02438	education years	0.098 [−0.05, 0.24]	0.18	DD (Childhood
					fluid intelligence	0.1 [−0.1, 0.302]	0.33	overgrowth)
					BMI	−0.014 [−0.16, 0.129]	0.85	
					height	0.059 [−0.08, 0.202]	0.42	
*RNF135*	dbSNP: rs121918161	chr17: 29324307 T>C	GenBank: NM_032322:c.C727T:p.Q243X	0.00215	education years	0.341 [−0.11, 0.79]	0.14	DD (macrocephaly,
					fluid intelligence	0.16 [−0.41, 0.726]	0.58	macrosomia,
					BMI	0.177 [−0.27, 0.626]	0.44	facial dysmorphism
					height	0.082 [−0.37, 0.532]	0.72	syndrome)
	Affx-80285705	chr17: 29325809 G>GC		0.05265	education years	0.02 [−0.08, 0.115]	0.68	
					fluid intelligence	−0.097 [−0.23, 0.032]	0.14	
					BMI	0.017 [−0.08, 0.113]	0.72	
					height	0.03 [−0.07, 0.125]	0.54	
*TGIF1*	dbSNP: rs202123354	chr18: 3452067 A>G	GenBank: NM_170695:c.G90A:p.W30X	0.01241	education years	0.023 [−0.23, 0.278]	0.86	DD (holoprosencephaly)
					fluid intelligence	0.169 [−0.2, 0.539]	0.37	
					BMI	0.134 [−0.12, 0.389]	0.30	
					height	0.053 [−0.2, 0.308]	0.68	

Classification of likely pathogenic variants in maturity-onset diabetes of the young (MODY) and developmental disorders (DD) from UKB. Abbreviations are as follows: UKB = UK Biobank, RSID = Reference SNP cluster ID, HGVS = Human Genome Variation Society, MAF = minor allele frequency, CI = confidence interval, BMI = body mass index, DD = developmental disorder.

### Refuting Previous Disease Associations

For the other three DD variants, our findings are not consistent with the genes’ causing a dominant DD via haploinsufficiency. First, there was no association between a frameshift variant in the middle of *COL4A3* (MIM: 120070)*—*where pathogenic variants are thought to cause a rare dominant form of Alport syndrome (MIM: 104200) (as well as benign familial hematuria [MIM: 141200])^66^, ^67^—and albumin creatinine ratios, hearing, or any of the development traits in UKB. Similarly, there was no association between either stop-gain or frameshift variants in *RNF135* (MIM: 611358) —where haploinsufficiency is thought to cause macrocephaly, macrosomia, and facial dysmorphism syndrome^68^ (MIM:614192)—with any development traits in UKB. In both cases, given the high-quality genotyping of these variants in UKB and a lack of association with any clinically relevant traits, together with a pLI of zero for both genes, the age of the original publications, and the lack of enrichment of *de novo* mutations within the DDD study,^33^ we suggest that haploinsufficiency in these genes is not a cause of a severe DD.

## Discussion

Previous studies have been unable to analyze rare variants in sufficiently large population-based studies to establish pathogenicity and lower bounds for penetrance. Large population cohorts such as UKB provide an opportunity to investigate the relationship between genes and disease. However, the absence of genome-wide sequencing data has thus far minimized the impact of UKB in the rare disease community. We have established a method, using combined intensity plots for individual variants across all genotyping batches, for evaluating the analytical validity of rare variants genotyped by microarray. Although we initially tried to examine variant cluster plots for each batch separately, as recommended by UKB, this proved impossible because of the rarity of most clinically important variants. MAF was an extremely good predictor of the likelihood that a variant would be genotyped well by the UKB arrays (Figure 1). At MAF > 0.005% (∼50 heterozygous individuals out of 500,000 in UKB) the FPR was ∼7%, and most variants were well genotyped, but the FPR was ∼60% at MAF > 0.001% (∼10 heterozygous individuals), and we classified all variants at MAF < 0.0005% (∼5 heterozygous individuals) as being low quality. This has important implications for epidemiological research carried out uncritically with these data. Although many rare variants in UKB are well-genotyped with the arrays, the rarer the variant, the more likely it is to be poor quality and therefore yield false associations.

A limitation of our work is that we did not attempt to confirm the variants by using an independent assay. However, most researchers using data from UKB will be similarly unable to attempt independent variant confirmations, and thus a method for evaluating the genotyping quality of rare variants directly from the data has widespread utility. The validity of our method is supported by our ability to replicate numerous previous findings of well-known, clinically important variants classified as pathogenic in ClinVar (Table 2 and Table S3, plus additional well-established associations for variants where MAF > 1%). In addition, our analyses of likely pathogenic variants in two disease subtypes (MODY and DD) were independent of any potential biases or misclassification errors associated with ClinVar, and the findings were consistent with our prior expectations. We expected there to be a small number of individuals in UKB with monogenic subtypes of diabetes, and we found two pathogenic variants that were associated with appropriate traits in UKB (Table 2); we were thus able to lower the previous penetrance estimate for a pathogenic variant in *HNF4A* (Figure 2). In contrast, we did not expect there to be any instances of severe DD, in light of the rarity of the condition, the relatively senior age of the UKB population, and the inherent challenges of obtaining consent from individuals with severe DD to be added to population biobanks.^69^ We therefore believe that the PTVs identified in dominant DD genes in UKB are benign (Table 3) and are confident in refuting previous associations between haploinsufficiency in *RNF135* and *COL4A3* and dominant DD (note that this refutation has no bearing on the asserted relationship between the latter and either recessive DD or alternative mechanisms of disease).

In this study, we have shown that population genetic data can be used for estimating lower bounds for the effect size and penetrance of pathogenic, disease-causing variants and refined our understanding of the links between rare variants (MAF <1%) and monogenic diseases. Performing a similar analysis on very rare variants (MAF <0.001%) will require large-scale sequencing data rather than genotyping arrays. Although population-based studies will be biased in the opposite direction from clinical studies, i.e. towards healthy individuals, they are nonetheless crucial for informing minimum and age-dependent penetrance estimates, interpreting incidental or secondary findings from clinical testing, and informing direct-to-consumer genetic testing. At this point, we are left with some fundamental conceptual questions about the nature of “monogenic” disease. When should variants exhibiting reduced penetrance—a term frequently used in the diagnosis of rare genetic disease—be called risk or susceptibility factors, terms generally used in the study of common disease? When should a gene-disease relationship be termed variable expressivity rather than normal variation? Should “pathogenic” be reserved only for highly penetrant variants that cause a tightly defined disease entity, or can it apply to any variant associated, however weakly, with a clinically relevant phenotype? As genome-wide sequencing becomes widely used in routine clinical practice, research cohorts, and direct-to-consumer testing, understanding this spectrum will become both increasingly important and tractable.

## Declaration of Interests

The authors declare no competing interests.

## Web Resources

DiabetesGenes, http://www.diabetesgenes.org
Evoker Lite, https://github.com/dlrice/evoker-lite
Gene2Phenotype, https://www.ebi.ac.uk/gene2phenotype
Online Mendelian Inheritance in Man, http://www.omim.org
UK Biobank Dataset, https://biobank.ctsu.ox.ac.uk/

## Supplemental Data

Document S1. Figures S1–S3

Table S1. Curated Traits Included from UKB

Table S2. List of 4,585 Putative Pathogenic Variants Evaluated

Table S3. Additional Information about Pathogenic Variants Associated with Disease

Document S2. Article plus Supplemental Data
