# Supplementary material for: Assessing the Pathogenicity, Penetrance, and Expressivity of Putative Disease-Causing Variants in a Population Setting
Source: Am J Hum Genet. 2019 Jan 18;104(2):275–86. doi: 10.1016/j.ajhg.2018.12.015 (PMC6369448; doi:10.1016/j.ajhg.2018.12.015)

The American Journal of Human Genetics, Volume *104*

## Supplemental Data

# Assessing the Pathogenicity, Penetrance, and Expressivity of Putative Disease-Causing Variants in a Population Setting

**Caroline F. Wright, Ben West, Marcus Tuke, Samuel E. Jones, Kashyap Patel, Thomas W. Laver, Robin N. Beaumont, Jessica Tyrrell, Andrew R. Wood, Timothy M. Frayling, Andrew T. Hattersley, and Michael N. Weedon**

## SUPPLEMENTARY FIGURES

**Figure S1. Combined cluster intensity plots.**
Intensity plots combined across all batches are shown for five variants, all with a UKB MAF = 0.004%. The clustering quality of heterozygous variants was manually assessed and ranked from 1-5. **(a)** Score 1 = poor quality, no discernible separate clusters; **(b)** Score 2 = poor quality, no discernible separate clusters but noisy data; **(c)** Score 3 = unclear/uncertain; **(d)** Score 4 = good quality, clearly separable clusters but noisy data; **(e)** Score 5 = good quality, clear separation between clusters.

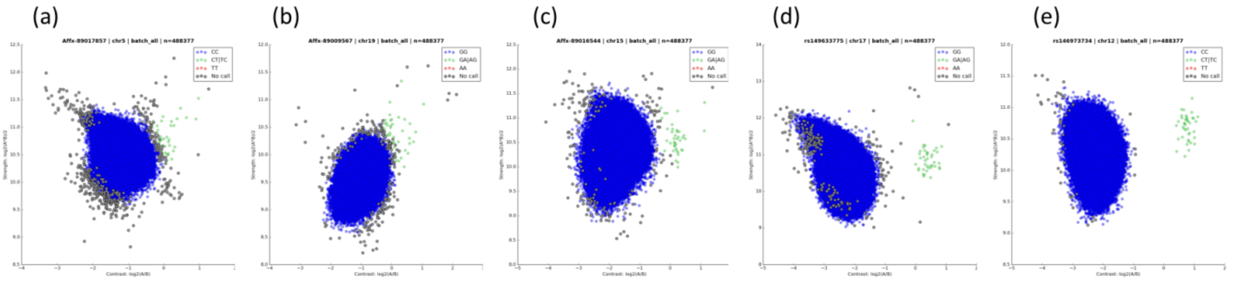


**Figure S2. Comparison of quality scores between two independent scorers.**
Two scientists independently scored the quality (from 1-5, see Figure 1) of combined cluster plots for 750 variants. The $R^2$ between their scores was 0.8, and there was a 95% agreement in low quality (score=1 or 2) versus high quality (score=4 or 5) variants.

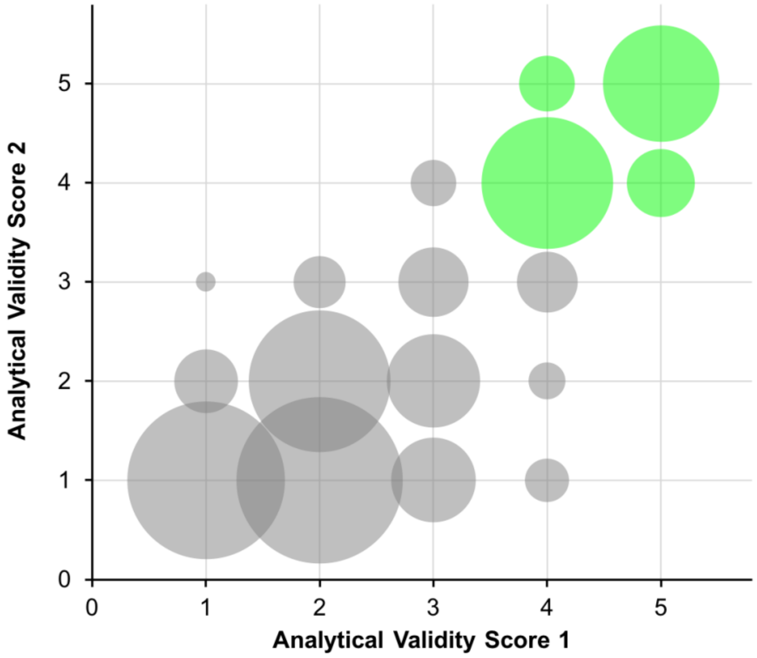

**Figure S3. Flowchart of included variants.**

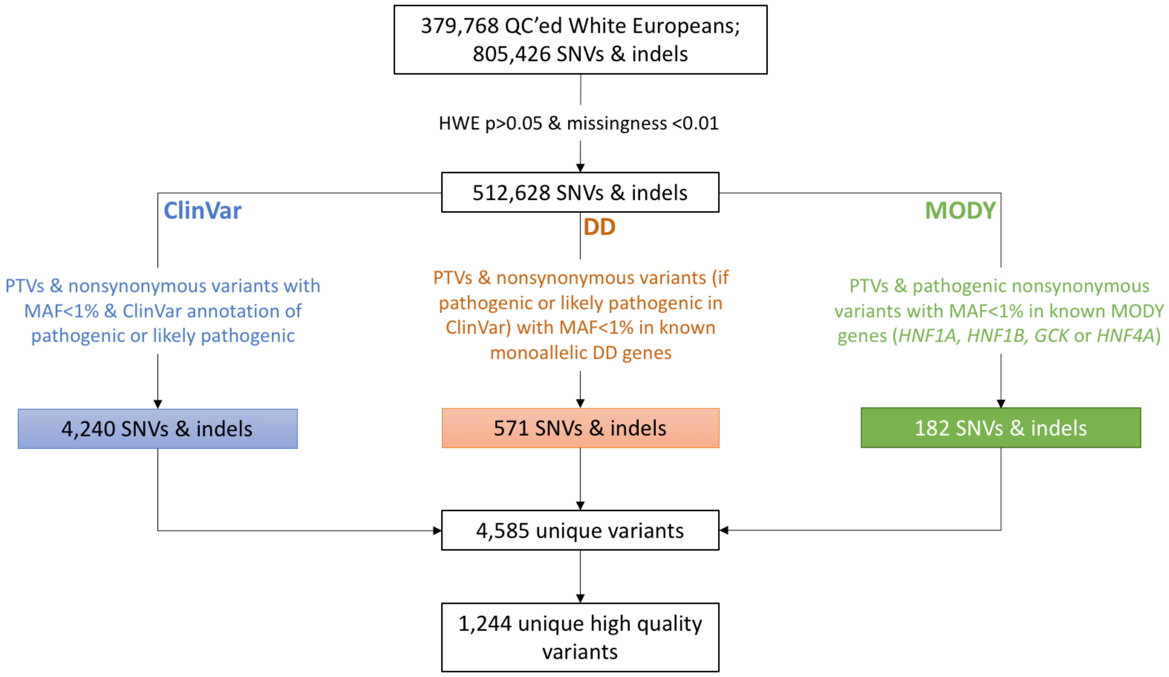

Supplement: Document S1. Figures S1–S3 [file mmc1.pdf]
